# Nurses’ and patients’ perceptions of physical health screening for patients with schizophrenia spectrum disorders: a qualitative study

**DOI:** 10.1186/s12912-024-01980-3

**Published:** 2024-05-11

**Authors:** Camilla Långstedt, Daniel Bressington, Maritta Välimäki

**Affiliations:** 1 University of Turku, Faculty of Medicine, Department of Nursing, Kiinamyllynkatu 10, Medisiina B, Turku, 20520 Finland; 2 Professor in Mental Health, Faculty of Health, Charles Darwin University, Casuarina, Australia; 3 Faculty of Nursing, Chiang Mai University, 110/406 Inthawaroros Road, Sri Phum District, Chiang Mai, Thailand; 4 University of Helsinki, School of Public Health, Helsinki, Finland

**Keywords:** Schizophrenia spectrum disorder, Severe mental illness, Physical health, Screening, Monitoring, Interview, Focus group, Qualitative

## Abstract

**Background:**

Despite worldwide concern about the poor physical health of patients with schizophrenia spectrum disorders (SSD), physical health screening rates are low. This study reports nurses’ and patients’ experiences of physical health screening among people with SSD using the Finnish Health Improvement Profile (HIP-F) and their ideas for implementation improvements.

**Methods:**

A qualitative exploratory study design with five group interviews with nurses (*n* = 15) and individual interviews with patients with SSD (*n* = 8) who had experience using the HIP-F in psychiatric outpatient clinics. Inductive content analysis was conducted.

**Results:**

Two main categories were identified. First, the characteristics of the HIP-F were divided into the subcategories of comprehensive nature, facilitating engagement, interpretation and rating of some items and duration of screening. Second, suggestions for the implementation of physical health screening consisted of two subcategories: improvements in screening and ideas for practice. Physical health screening was felt to increase the discussion and awareness of physical health and supported health promotion. The HIP-F was found to be a structured, comprehensive screening tool that included several items that were not otherwise assessed in clinical practice. The HIP-F was also considered to facilitate engagement by promoting collaboration in an interactive way. Despite this, most of the nurses found the HIP-F to be arduous and too time consuming, while patients found the HIP-F easy to use. Nurses found some items unclear and infeasible, while patients found all items feasible. Based on the nurses’ experiences, screening should be clear and easy to interpret, and condensation and revision of the HIP-F tool were suggested. The patients did not think that any improvements to the HIP-F were needed for implementation in clinical settings.

**Conclusions:**

Patients with schizophrenia spectrum disorders are willing to participate in physical health screening. Physical health screening should be clear, easy to use and relatively quick. With this detailed knowledge of perceptions of screening, further research is needed to understand what factors affect the fidelity of implementing physical health screening in clinical mental health practice and to gain an overall understanding on how to improve such implementation.

## Background

The physical health state of people diagnosed with schizophrenia spectrum disorder (SSD) is a global problem [1]. Typically, poor physical health results from a range of issues, including the impact of psychiatric symptoms on health behavior, adverse effects of prescribed medication, difficulties observing physical health concerns, lifestyle, diagnostic overshadowing, and patient unwillingness to report health problems [2, 3]. These factors may lead to obesity, metabolic syndrome, coronary vascular disease, diabetes, hypertension, or cancer [4–6]. High rates of infectious diseases such as hepatitis and HIV [7] and COVID-19 [8] have also been reported in patients with SSD. As an outcome of physical health issues, physical comorbidity is associated with psychiatric readmission [9] and high treatment costs. In Finland, the total healthcare costs caused by schizophrenia are approximately 700–900 million euros per year, mostly as a result of inpatient treatment costs [10]. Due to poor physical health, the life expectancy of persons with schizophrenia is approximately 20 years less than that of the general population [11, 12]. Therefore, it is crucial that physical health screening is conducted regularly for patients with SSD. Improving regular screening helps to support earlier detection of risk factors that can, without detection and intervention, have deleterious effects on the physical health of patients with SSD [10].

Several international clinical guidelines have recommended how physical health screening for patients with SSD should be conducted [10, 13–16]. According to guidelines persons with SSD who have been prescribed antipsychotic medication should have annual health checks focusing on full blood count, lipids, plasma glucose, prolactin, blood pressure, urea, electrolytes, liver function tests, weight, waist circumference measurement and electrocardiogram examination (ECG) [16]. Being aware of patients’ lifestyle habits, including smoking and use of other substances [10, 13, 15] is important for directing appropriate behavioral interventions to promote healthy lifestyles. In addition, a variety of screening instruments have been developed to assess physical health among people with SSD. Lamontagne-Godwin et al. [17] identified in their systematic review 44 intervention studies aiming to increase access to or uptake of physical health screening. Examples of monitoring tools in the included studies were Physical Health Check (PHC) [18]; physical health monitoring sheet [19]; systematic computerized cardiovascular health screening [20]; the Metabolic Syndrome Screening Tool (MSST) [21]; quality improvement (QI) [22] to increase rates of metabolic syndrome screening and the Health Improvement Profile (HIP), which is a comprehensive nurse-led profiling tool that assesses physical health risks, identifies unhealthy lifestyle behaviors, and provides associated recommended actions for health promotion [23]. Despite the abundance of available instruments, physical health screening is still poorly implemented in clinical mental health services [24, 25].

To better understand this rationale for poor physical health screening, a quantitative study in Uganda [26] showed, that more than 75% of 28 nurses had a positive attitude towards metabolic screening and associated interventions. The same study reported that more than 50% of nurses were confident in providing physical activity and smoking cessation advice and nutritional counseling. However, 57% stated that their heavy workload prevented them from doing health screening. Voort et al. [27] reported in their qualitative study in Netherlands, that most nurses perceived physical health screening to be an important part of their professional role, but identified a discrepancy between their perceptions and actual clinical practice. Happell et al.’s qualitative study [28] reported in Australia that although nurses recognize their responsibility with respect to the physical health of patients with severe mental illness, they experienced factors such as staff shortages and lack of knowledge that prevented them from conducting screening properly. Further, Mwebe [29] reported in his UK study that nurses shared a clear commitment regarding their role in physical health screening in mental health care settings. Four themes emerged as follows: features of current practice and physical health monitoring; perceived barriers to physical health monitoring; education and training needs; and strategies to improve physical health monitoring. In the UK, Butler et al.’s qualitative study [30] revealed that patients varied in their awareness of the association between mental and physical health, but were engaged in physical health screening.

Moreover, Bressington et al. [31] revealed in their qualitative study, that nurses working in Hong Kong psychiatric care settings found the HIP (the Health Improvement Profile) to be comprehensive and perceived positive changes in their patients’ wellbeing, for example, by increasing motivation for patients to improve their health. HIP was developed to increase patient engagement in screening their physical health in collaboration with a nurse [32]. Earlier studies in the UK [33], Hong Kong [34], and Thailand [35] have reported patient acceptability and clinical utility of the HIP in identifying health risks where interventions are needed. These findings show that HIP may be feasible in engaging patients in discussions about physical health and in identifying areas of health risk [34, 35]. Although Hardy et al. [33] found support for the usability of the HIP in clinical practice in a study in the UK, a subsequent RCT study conducted in the UK revealed that nurses found the use of the HIP unfeasible in a clinical setting due to its length [36]. In contrast, nurses in Hong Kong [31] found the HIP to be acceptable, feasible, and potentially useful in clinical practice. In Finland, our validation study of the Finnish Health Improvement Profile (HIP-F) supported this finding by detecting 399 areas of health and health behavior risk in a sample of 47 patients [37].

Previous international studies have only reported nurses’ and patients’ general attitudes toward health checks without detailed perceptions of the importance of comprehensively assessing different health parameters together with ideas for improvements. Implementation of physical health screening is influenced by services users’ perceptions and experiences. It is of paramount importance to involve potential users in the design and implementation of new procedures [38], and thus, when developing physical health screening for patients with SSD, the perceptions of both nurses and patients are vital [38, 39]. Reconciling patients’ and nurses’ perceptions of physical health and its screening is an important step in promoting collaborative care and improving physical health screening rates [40]. Little detailed information is known about how nurses and patients perceive physical health screening; particularly, the assessment target areas and parameters, and how would nurses and patients improve screening so that it is more likely to regularly conducted in clinical practice. No previous studies have aimed to understand detailed perceptions and ideas for improvements of physical health screening by combining both nurses’ and patients’ perspectives using qualitative methods. The contrasting results regarding HIP instrument highlight that the acceptability and feasibility of HIP might be culturally and clinically context specific, and more research on patients’ and nurses’ perceptions of HIP in clinical practice is needed. To fulfill this knowledge gap, the current study sought to explore nurses’ and patients’ perceptions of physical health screening using the HIP-F profile as an example of physical health screening among patients with SSD in psychiatric settings in Finland and identify possible areas for improvement in the HIP-F tool and screening procedures.

## Methods

### Aim

The aim of this study was to explore (1) nurses’ and patients’ perceptions of physical health screening using the HIP-F profile as an example and (2) possible areas of improvement for implementation of physical health screening among patients with SSD in psychiatric settings in Finland. The information can be used to identify possible areas to be improved regarding implementation of systematic physical health screening activities as a part of treatment process among patients with SSD.

### Study design

A qualitative exploratory study design, with focus group interviews for nurses and individual interviews for patients, was used to gain a better understanding of the real-life experiences of the study participants [41, 42]. The qualitative exploratory design was appropriate for defining the terms of the research problem and to gain background information on a topic that little is known about [42, 43]. For nurses, focus group interviews were used not only as a way of obtaining individual answers but also with the group interaction of participants to allow participants to explore and clarify individual and shared perspectives of specific phenomena in an open and flexible way [43, 44]. For patients, individual interviews were chosen to receive deep insight into the respondent’s personal thoughts and feelings but also to ensure privacy, confidentiality, and a comfortable atmosphere, with concern for the vulnerability of patients with SSD [13] Moreover, individual interviews for patients were conducted to pursue personal disclosure and with consideration of the possible cognitive disabilities, such as attention and memory issues of patients with SSD [45]. Despite the potential for cognitive dysfunction, there is several benefits, such as receiving patients’ perspectives affecting engagement, involving consumers in developing interventions [46]. An exploratory approach was selected to obtain more detailed descriptions of the experiences of the participants. With this approach, we aimed to identify the phenomenon by using open-ended questions to allow nurses and patients to freely express their perceptions so that we could perform an inductive content analysis on the data without any theoretical framework or previously produced codes and categories [42, 47].

We adhered to the consolidated criteria for reporting qualitative studies (COREQ) [48] when reporting the current study.

### Setting

The study was conducted in five psychiatric outpatient clinics in Southern Finland. These clinics were selected because they offer a desirable representativeness of the study population, being part of the largest hospital area in Finland with a population of approximately 460,000 inhabitants [49]. The clinics provide mental health care for approximately 2,300 patients who have been diagnosed with a range of schizophrenia spectrum disorders (F20–29) [50]. The clinics provide both crisis and long-term mental health care and focuses on recovery and rehabilitation provided by multidisciplinary teams (psychiatrists, social workers, mental health nurses) as well as counseling and psychiatric examinations [51]. The patients’ frequency of attendance at the clinics depends on their individual treatment plan.

### Sampling

For nurses, a purposive sampling method was used to recruit enough participants and generate enough rich data to understand the studied phenomenon [52]. All 47 nurses who had previously been asked to use the HIP-F to assess the physical health of their patients, were invited to join the focus group interviews. These nurses had diverse backgrounds of various ages, education, and length of working experience and had the potential to provide relevant and diverse data pertinent to the research question [53, 54]. The inclusion criteria for nurses were that they had professional education (registered nurse, mental health nurse), that they had permanent or long-term temporary employment and that they were currently working in mental health clinical practice as a patient’s primary nurse in coordinating and providing care. The exclusion criterion was being a nursing student. We aimed to sample a total of 5 focus groups, one from each study clinic, with 6–10 nurse participants in each focus group, which is close to an optimal size in focus groups to promote discussion. The sample size estimation was based on previous literature suggesting that at least four focus groups would be sufficient to identify new issues (code saturation), but more groups may be needed to completely understand these issues (meaning saturation)” [52].

For patients, a purposive sampling method was used to recruit eligible participants for the individual interviews. To be eligible to be invited to participate, the patients needed to have a diagnosis of a schizophrenia spectrum disorder, to have been treated as an outpatient in a clinic and to have been previously targeted for physical health screening with the HIP-F to elicit feedback on their experiences and perceptions [55]. We aimed to recruit 10 patients for the individual interviews since this number of interviews in qualitative content analysis was believed to allow us to reach a saturation of themes [56]. The inclusion criteria for patients were a minimum age of 18 years, being treated in outpatient clinics, having the ability to understand and speak Finnish, and a diagnosis of schizophrenia or another schizophrenia spectrum disorder F20-29 (ICD10) [50]. The exclusion criteria were having an acute psychosis or a very disturbed mental state, where participation would distress the patient or put nurses at risk.

### Interview questions

Participants were asked to give their responses to open-ended questions, which focused on physical health screening with the HIP-F. The original HIP instrument, a physical health screening tool, was developed in the UK [32] and validated in Finland [37]. The HIP is a 27-item (28 for females) gender-specific profiling tool focusing on physical health and health behavior items (see Table 1). It enables nurses and patients to work together to assess physical health among patients with SSD. Health items (e.g., smoking status) are evaluated by categorizing them as green (e.g., nonsmoker) or red (e.g., passive smoker/smoker) depending on the result. If the health item is assessed as red, recommended actions (e.g., advice that all smoking is associated with health risks, refer to smoking cessation service) can be selected to produce a health care plan. The HIP is intended to be completed at least annually, which is the recommended frequency of screening for patients with SSD [12, 50]. This assessment together with regular discussions with a nurse familiar with the patient might decrease barriers, for example, in talking about sensitive topics [17]. In this study, the perceptions of recommended actions have not been reported because we aimed to study only the nurses’ and patients’ experiences and perceptions of the screening procedure.

**Table 1:** HIP-F items

BMI	Feet
Waist circumference	Smoking status
Pulse	Exercise
Blood pressure	Alcohol intake
Temperature	Diet: 5 portions a day
Liver function tests (in last 3 months)	Diet: fat intake
Lipid levels	Fluid intake
Glucose	Caffeine intake
Cervical smear (female)	Cannabis use
Prostate and testicles (male)	Safe sex
Menstrual cycle (female)	Urine
Sleep	Bowels
Teeth	Sexual satisfaction
Eyes	Breast self-examination (female and male)

The interview questions were based on the process observation method used in a UK-based cluster-randomized controlled trial with HIP [36] and a qualitative descriptive HIP study in Hong Kong [31]. An overview of the open-ended questions is as follows:

- How did you experience the physical health screening with the HIP-F?
- What did you think about the physical health screening?
- Which elements of assessing physical health with the HIP-F did you find most and least feasible?
- How long did it take to complete the HIP-F?
- What improvements could be made to physical health screening?

### Recruitment

First, for potential nurse participants, one researcher (CL) provided information sessions about the study to each study clinic twice via Teams meetings. Information was given about the rights, voluntariness and confidentiality, and purpose of the study, as well as the process and the risks and benefits of participating. The main risk of participating would be the time spent participating in the research. The research would not produce immediate benefits for the nurse participants, but it would give an opportunity to influence the improvement of the usability of the HIP-F profile by giving feedback and suggestions for changes. Nurses were informed about what to expect from the focus group interviews to increase the likelihood of honesty. Participants also received written information by email before they gave their written informed consent. Nurses expressed verbally their possible desire to participate to the researcher during the information sessions and the researcher collected the consent form from the participating nurses from the study clinics at the agreed time. Of the 47 eligible nurses, 16 agreed to participate. However, one of the agreed nurses withdrew before the interview. The researcher regularly visited the study clinics (once a week), obtained informed consent from participants, and contacted the participating nurses to agree on dates for the focus groups.

Second, patients were recruited by nurses during their regular meetings in study clinics after they had been screened with the HIP-F. Nurses informed patients about the voluntariness and confidentiality as well as the purpose of the study, the process, and the risks and benefits of participating. There would be no direct benefit to the patients from participating, and no other disadvantages than the time spent on the interview. It was deemed unlikely that patient participants would experience any distress as a result of participating. Patients were given both oral and written information from nurses that participation or refusal to participate would affect their treatment in the clinic or their relationship with the clinical staff. Since cognitive problems may be associated with SSD [13], we aimed to ensure that each nurse would recruit familiar patients using an assessment of their cognitive ability and their capacity to give informed consent for participation [13, 45]. Altogether, eight patients participated and gave their informed consent to a nurse who informed the researcher of the patient’s participation. The researcher contacted the patients to agree on dates for the individual interviews.

### Data collection

Interviews were conducted using a semi-structured format to encourage participants to talk about issues that would answer the research question [57, 58]. Before the interviews, participants gave their background information regarding gender and age. Nurses were also asked about their education and work experience in mental health care. The researcher guided the participants in the focus group interview and encouraged them to interact with each other [59]. The focus groups were preexisting work groups from clinics, and this facilitated open discussion and interaction with shared experiences in a comfortable and familiar setting [57].

All interviews were conducted between October and December 2022 by one female researcher (CL), a registered nurse (PhD student) with a long working experience with patients with SSD, who was working as a nurse manager in another unit. The researcher knew one nurse participant from an earlier HIP validation study. Participants knew that the research was a part of the researcher’s PhD study. Only the researcher and study participants were present during the interviews. Consent for recording was obtained from all participants. No pilot interviews were used. Altogether, four group interviews with nurses with two to six participants in each interview were conducted. One nurse was individually interviewed because the other consenting participant withdrew. Four of the nurses’ interviews occurred in clinic meeting rooms, and one was held via Microsoft Teams meeting. For the patients, eight individual interviews were conducted: seven by phone and one in Microsoft Teams meeting after the researcher called the patient with Microsoft Teams application. These approaches were chosen so that the subjects would experience as little harm as possible from participating in the study, for example an extra visit to the research outpatient clinic. When conducting the interviews, the current restrictions due to the COVID-19 pandemic also had to be taken into account. The duration of the interviews with nurses varied from 25 to 56 min, and the patients’ interviews lasted from 8 to 32 min. During the first two interviews, the researcher evaluated whether the questions were clear and relevant according to the information received. As no participant asked for clarification and the data were considered relevant, the questions were used in all interviews. No field notes were made during the focus group interviews and the patient interviews, but records were made about observations of nonverbal responses and reflections in the nurses’ interviews as soon as possible after each interview [60].

### Data analysis

The data analysis was conducted concurrently with the interviews. The interviews, original transcriptions, and overall data analysis were in Finnish. An inductive content analysis method for audio-recorded interviews was chosen since there are no previous qualitative studies on the topic in Finland [61]. When conducting exploratory research in an area where little is known, content analysis might be suitable for the reporting of general issues in the data [62]. Furthermore, content analysis was well suited for analyzing our study topic, which is a sensitive, important, and multifaceted phenomenon of nursing [58, 63]. Since we aimed to generate complementary perceptions and an enhanced understanding of the phenomenon, focus group and individual interview data were combined for analysis [64]. All interviews were transcribed in Word 2021 and analyzed using the five-step method by Graneheim and Lundman [65]. This approach enabled a systematic, reliable, and valid data analysis [58], which was led by research questions [66]. No software was used for coding in the analysis. First, all interviews were transcribed verbatim by one researcher (CL). Second, the researcher initially familiarized herself with the data through multiple careful readings of the transcripts to gain an understanding of the whole. Third, a sentence was selected as an analysis unit. Fourth, the text was distributed into meaning units, which were further condensed into sentences, and the condensed meaning units were abstracted and labeled with a code. Fifth, all 18 codes identified from the data were compared with each other for similarities and differences and sorted into six subcategories. The tentative categories were discussed between all authors and revised. A process of discussion and reflection resulted in an agreement on how to sort the codes.

Finally, the subcategories that were similar in terms of meaning and content were sorted into two main categories. Quotations from study participants were translated into English by one author (CL), checked by another bilingual researcher (MV) for equivalent meaning, and presented to illustrate the results (N as nurse, P as patient). From the first to the third nurses’ interview a total of 13 codes were added, and no further new codes were developed after the fourth interview. Based on code identification (88% of codes had been identified), code prevalence (90% of high-prevalence codes were identified) and codebook stability (94% of codebook changes were made), code saturation was reached after four interviews. Meaning saturation was reached at the last interview in which a new dimension of the code was identified. [52.] From patients’ interviews, code saturation was reached after the fourth individual interview and meaning saturation was achieved after the eighth interview as the repetition of content became obvious [52, 67]. Examples of meaning units and codes are presented in Table 2.

**Table 2:** Examples of meaning units, codes, subcategories, and main categories

Meaning units	Codes	Subcategories	Main categories
Screening is important because it includes health parameters that would not be assessed otherwise	Importance	Comprehensive nature	Characteristics of the HIP-F
The questionnaire could be condensed	Condensation	Improvements in screening	Suggestions for the implementation of Physical health screening

## Results

### Demographic characteristics of study participants

A total of 15 nurses participated in the study (11 females and four males). The distribution of nurses was as follows: in the first interview there were three males and one female; in the second group there were two females; in group three there was one male and one female; the fourth interview contained one female and in the fifth interview there were six female nurses. The ages of the participants varied between 43 and 61 years, with a mean age of 49.47 years (SD 5.99). The majority were registered nurses. The length of their work experience in mental health nursing varied from one and a half years to 38 years, with a mean working experience of 21.73 years (SD 8.18). Among the patients, seven females and one male participated in the study. The ages of the participants varied between 21 and 65 years, with a mean age of 43.87 years (SD 17.27). The demographic characteristics of the study participants are presented in Table 3.

**Table 3:** Characteristics of study participants

	Nurses *n*= 15	Patients *n*=8
**Age**		
20–30 years	-	3 (37.5%)
30–40 years	-	- (-)
40-50 years	8 (53.4%)	1 (12.5%)
50–60 years	5 (33.3%)	3 (37.3%)
> 60 years	2 (13.3%)	1 (12.5%)
**Gender**		
Female	11 (75.0%)	7 (87.5%)
Male	4 (25.0%)	1 (12.5%)
**Professional background**		
Registered nurse	12 (80.0%)	-
Mental health nurse	3 (20.0%)	-
**Length of experience in mental health nursing**		
< 10 years	1 (6.7%)	-
10–20 years	2 (13.3%)	-
20–30 years	10 (66.7%)	-
> 30 years	2 (13.3%)-	-

### Nurses’ and patients’ perceptions of physical health screening with the HIP-F and suggestions for improvement of screening in psychiatric settings

Both nurse and patient participants perceived physical health screening among patients with SSD to be important and the screening with HIP-F as an example screening tool to be comprehensive, but also highlighted some areas for improvement for conducting screening in psychiatric settings. Two main categories were identified from the analysis. First, the characteristics of the HIP-F were divided into subcategories: *comprehensive nature*, *facilitating engagement*, *interpretation and rating of some items*, and *duration of screening*. Second, suggestions for the implementation of physical health screening consisted of two subcategories: *improvements in screening* and *ideas for practice*. The summary of codes, subcategories and main categories is presented in Table 4.

**Table 4:** Codes, subcategories and main categories

Codes	Subcategories	Main categories
Importance Comprehensiveness Feasible items	Comprehensive nature	Characteristics of HIP-F
Positive experience Regularity Interactivity Increased discussion of health Increased information on health Possibility for health promotion	Facilitating engagement	Characteristics of HIP-F
Arduous Ambiguity Infeasible items	Interpretation and rating of some items	Characteristics of HIP-F
Broadness Time consuming	Duration of screening	Characteristics of HIP-F
Condensation Revision of assessment	Improvements in screening	Suggestions for the implementation of physical health screening
Educational needs Filling in the HIP-F beforehand	Ideas for practice	Suggestions for the implementation of physical health screening

### Characteristics of the HIP-F

#### Comprehensive nature

The patients and nurses considered the HIP-F tool to be important, structured and able to comprehensively evaluate physical health. Patients found alcohol intake, activity and smoking status to be extremely important to assess among patients with SSD and expressed that it was the first time nurses had asked about several of the important items in HIP-F, including urine, caffeine intake and sexual satisfaction. Participants stated that the HIP-F includes several items, such as urine, caffeine intake, feet, and sexual satisfaction, which would not be assessed otherwise. Nurses expressed that in clinical practice a range of different nurses evaluate patients’ physical health parameters dependent on the clinical setting, however there is a current lack of appropriate structured, comprehensive screening tools. Based on the experiences of most nurses and all patient participants, all HIP-F items were considered feasible. Most of the nurses expressed that all items assessed with laboratory tests as well as body mass index (BMI), waist circumference, diet, activity, alcohol intake, teeth, smoking status, eyes, and caffeine intake were particularly feasible in physical health screening. However, despite the importance and feasibility, some HIP-F items were considered potentially challenging to talk about (e.g., sexual satisfaction) because of their sensitive nature.

*The items are kind of structured here, because there is a lot, a lot of things we are asking, but they are being asked scattered in different situations, in different phases…yes, the comprehensiveness is good. (N5)*

*Yes, there was the alcohol intake and smoking status and activity, they seemed essential. (P6)*

*Yeah, well, it could be that for some people, the things related to their own sexual life are the same, which they don’t necessarily want to discuss. (P5)*.

#### Facilitating engagement

All participants found the physical health screening with HIP-F to be an overall positive experience. Patients were fully aware of the significance of the relationship between physical health and mental health and were happy to have their physical health assessed. All study participants stated that physical health monitoring with the HIP-F on an annual basis is a relevant timespan for regular health checks. Based on the nurses’ and patients’ experiences, the participants felt that conducting the HIP-F together in an interactive way facilitates engagement with physical health screening and health promotion. The participants described this working model to be more desirable, making health checks easier and enabling patients to have feedback on their state of health immediately. Furthermore, nurses stressed the importance of engaging patients with SSD in their own care, something that is supported with HIP-F screening. The patients and most of the nurses expressed that the screening increased discussion in general, and discussion about physical health between nurses and patients in areas that would otherwise not be discussed. According to the study participants, screening improved information, raised some thoughts and increased awareness of physical health and health behavior in general and particularly about the items that affect patients with SSD. Nurses experienced that especially with patients with SSD it is more beneficial to conduct the screening together during a discussion because of patients’ potential cognitive challenges. The participants described that screening helped to identify physical health illnesses which helped them to start adequate treatment for the patient. Based on the participants’ experiences, screening might motivate patients to increase their activity, support physical health, and strengthen already healthy life behaviors.

*Well, I wouldn’t mind if this assessment would be conducted once a year. (P5)*

*Yes, it is very good, especially with our psychosis patients, that we are engaging them in treatment, especially in somatic health. (N5)*

*Yes, it raised at least a little discussion about physical health. (P1)*

*In fact, we caught quite a hypertension disease, so that was the end of it. (N2)*.

#### Interpretation and rating of some items

Most of the nurse participants experienced HIP-F as arduous to conduct and challenging in screening, especially without routine. Nurses described the HIP-F to be too complicated and too precise and that some items were difficult to assess; for example, items pertaining to urine, fat intake (diet), five portions a day (diet), and activity were found to be difficult to assess. Nurses stated that the amount of urine passed is difficult to assess just by asking patients about it. Nurses also experienced that screening with HIP-F was too precise because nurses believed that their main work task is to evaluate mental health state, not physical health. Furthermore, some nurses were not familiar with the measurement units for some HIP-F items. However, patient participants expressed that HIP-F was easy to conduct. Moreover, nurses experienced that the HIP-F was ambiguous and partly difficult to interpret. Nurses described that some items, units of measure, and cutoff values were unclear; for example, the items for fluid, caffeine and alcohol intake, as well as the items concerning feet and urine, were overall experienced to be strange, and the significance of urine as an item remained unclear. Nurses stated that for some items, they could not find an adequate alternative to the cutoff values. This made most of the nurses consider the HIP-F to be ambiguous, which made conducting it frustrating. Some of the nurses experienced that the HIP-F also included items that were not feasible in physical health screening, such as safe sex, breast examination (men), body temperature, five portions a day (diet), caffeine intake, liver function, sexual satisfaction, BMI and feet check.

*Some items felt weird, perhaps I didn’t quite understand why these were being asked so precisely in a mental health care context. (N10)*

*I think one challenge was for example those…there are lipids or blood sugar, so, how was it, it’s quite a long time since you have done these…I had to check from the patient record how they are assessed in Finland, are they millimole or what, to find the congruent values and what are they then. (N1)*

*The item alcohol intake is weird, there is no alternative to choose if you don’t use alcohol at all. (N9)*

*It was a quite an easy questionnaire. Yes, it felt like that, and truly clear. (P6)*.

#### Duration of screening

Nurses found the HIP-F too broad and time consuming to be used in clinical practice in a psychiatric setting and not feasible to be implemented in Finnish mental health services. Nurses described that although physical health screening among patients with SSD is crucial and the HIP-F includes important health items, it is too long to be used in clinical practice. Even those nurses who were first motivated to conduct screening, did not continue screening with several patients when they found out how long the screening took. Nurses reported the heavy workload of caring for many patients and their main tasks in the mental health treatment setting. Nurses experienced that the screening with HIP-F took all the time from the scheduled appointment and no time was left for discussion about the mental health of the patients, so they decided to choose to assess possible psychotic symptoms or patients’ functioning ability. Furthermore, nurses described that during screening, it was found that some health parameters, for example, annual laboratory tests, had not been conducted on patients, even if they should have been conducted according to the clinics’ regular procedures, and this challenged and delayed the screening. Nurses stated that conducting the HIP-F screening takes from 45 to 60 min, which they felt was too much for patient meetings, especially if patients only seldomly have appointments. Some nurses experienced the HIP-F to be easy to use but still too time consuming. Whereas, some of the patients had been prepared for a longer assessment and expressed that the HIP-F screening was suitable in length.

*Well, I made one at the beginning, and when I noticed that it was arduous and how much time it takes, maybe that’s when the enthusiasm faded. (N9)*

*I’m guessing 45–60 min, I haven’t recorded it, but usually we have 45-minute appointments and sometimes it takes slightly more, and it took me the whole time to do it. (N10)*

*I was prepared for a longer questionnaire, but it wasn’t. (P5)*.

### Suggestions for the implementation of physical health screening

#### Improvements in screening

Nurses suggested lightening and condensing the content of the HIP-F. They described that screening could be shorter and that some HIP-F items could be left out. For example, one nurse expressed that asking about temperature in physical health screening is pointless unless the patient has a cold. However, some of the nurses and all patients felt that no improvements were needed in screening for implementation. Some nurses suggested that the cutoff values could be removed, and the items could remain just as a checklist for discussion. However, other nurses thought that the cutoff values should be retained in screening and that there was nothing to develop or leave out. In addition, some nurses stated that items, such as blood pressure, could be assessed numerically but that there could be additional space for open narrative text. On the other hand, some nurse participants expressed that the assessment might be ineffective without cutoff values. Furthermore, participants expressed that some items could be assessed differently. Nurses suggested that, for example, instead of asking patients about their amount of urine output, patients could be asked about hematuria, and instead of asking about teeth checks, patients could be asked if they are brushing their teeth regularly. One patient suggested that instead of assessing activity levels, patients could be asked what kind of activity they prefer. Another patient suggested that sexual satisfaction could be assessed more broadly, taking sexual diversity into account. In addition, nurses suggested that the layout and order of the items could be different: the green and red areas could appear in green and red on the HIP-F form, and a yellow area could be added. This was considered to be more effective in demonstrating to patients their physical health state and highlighting possible areas which should be improved, rather than just discussing about the results of the HIP-F screening. Nurses stated, that adding yellow areas in HIP-F would show patients that although the result is still in a healthy area, if no improvements are made, subsequent physical health problems are likely.

*I would remove temperature. It should be normal if you don’t have cold. (N12)*

*I wouldn’t directly remove anything. (N5)*

*Yes, I said that I could take all these cutoff values out of here and keep it just as a check list so these would be checked with a patient at least once a year. (N3)*

*However, if there were no cutoff values for activity, sleep and smoking, then… I think these traffic light systems would be good if you could get it in color so that if it is shown to the patient who you now have this in red, that you should probably do something about it. (N1)*

*Therefore, it could be three-part if there were the traffic light like you said just now, if it was the yellow light in between as well. (N4)*.

#### Ideas for practice

Most of the nurses expressed that the HIP-F includes basic physical health items and that conducting health screening with the HIP-F in clinical practice does not require any additional training. However, one nurse expressed that education for talking about sensitive topics, such as safe sex and sexual satisfaction with patients, is needed. Some of the nurses suggested that the HIP-F could at least partly be completed beforehand by the patient before their clinic appointment so the screening would not take too much time from the appointment. One nurse suggested that this could happen by using an electronic version (i.e., a software application) instead of a paper questionnaire, especially for younger patients with technical skills. In addition, some of the nurses suggested a separate, longer appointment for patients in the clinics for physical health screening.

*These are just basic things, there is no need for additional training. (N9)*

*Yes, some could be doing it in advance, and some would be that who you would measure the blood pressure or something together…I think it would be reasonable, that it would already be…the patient would have already filled it in beforehand as best they could and perhaps thought about these things in peace at home, so that would speed it up in the appointment. (N10)*.

## Discussion

As far we are aware, this study is the first study to explore perceptions among nurses and patients with SSD of physical health screening. We used the HIP-F profile as an example of a physical health screening tool. We aimed to identify possible areas for improvement in the tool and screening procedures. The study reveals several important aspects of how nurses and patients perceive physical health screening. At the same time, the HIP-F tool was also found to be arduous and time consuming, which led to recommendations on key improvements to the tool and physical health screening procedures.

Our study showed that nurses perceived physical health screening to be important [27, 68] and that they appreciated the comprehensive physical health screening with HIP-F [28, 31]. Nurses expressed that several HIP-F items were particularly feasible. Patients also found physical health screening beneficial in improving their awareness of physical health, which can potentially trigger health promotion conversations between nurses and patients [18, 28, 31]. Patients in our study were interested in and satisfied with having regular assessment of their health status [30, 33, 69–71]. Indeed, the theme ‘facilitating engagement’ was identified as a crucial factor for successful health screening in both nurses’ and patients’ data [26, 27, 30]. Our results are encouraging since previous studies have revealed that negative attitudes among nurses and a lack of support may restrict systematic health checks in mental health services [30, 31]. In some countries, for example Turkey [72], nurses have stated that patients are not interested in participating in health checks. Positive perceptions among nurses towards any new intervention, including physical health screening, are important in facilitating the integration of new practices into patient care [73, 74].

Some divergent perceptions were also found in nurses’ and patients’ perceptions in our study. Patients did not identify any infeasible or unclear items in their physical health assessment while nurses identified items regarding urine, caffeine intake, temperature, safe sex, or sexual satisfaction not meaningful or difficult to complete [37]. The finding regarding urine problems in patients with SSD is interesting as polydipsia may lead to water intoxication [75]. Patients with SSD are also 29 times more likely to get a urinary tract infection, which is a precipitating factor for acute psychosis [76, 77]. Sometimes nurses perceive their subjective clinical view as more crucial in assessing patients’ health status than using the objective results of a standard screening tool [78]. In the future, the core reason for this discrepancy should be explored to fully understand nurses’ avoidant behavior in conducting systematic health screening with patients. This is important because our current results may be contradictory with the reality. For example, although health screening was seen as an important task in patient care, the nurses complained that using HIP-F took too much time, which made them avoid patient health screening. For example, in the current study out of 47 nurses who had been asked to conduct HIP-F screenings with their patients, only 16 were willing to use the HIP-F screening tool and monitor their patients’ physical health. This finding is interesting as it highlights the benefit of collaboration between nurses and patients when conducting screening together, as reported in previous studies [35, 36, 79]. At the same time, nurses expressed that the screening process was unclear and difficult to follow [17, 29, 80]. To adopt healthy lifestyles, e.g. physical activity and nutrition, nurses should integrate improvement initiatives for patient physical health into daily practice by making small changes [71]. In this study, however, nurses perceived assessment of patient physical health using HIP-F as a separate task, which caused double recording in patients’ health records. This finding concurs with earlier studies that health screening is poorly implemented into mental health practice [24, 25].

In our study, nurses suggested condensation of the screening and revising the assessment with more culturally-understandable units of measurements. Item terminology should also be better suited into clinical practice [31]. To improve patients’ ability to understand the results of their health assessment, nurses suggested use of ‘a yellow traffic light’ as already used in the Chinese Health Improvement Profile (CHIP) [34]. Therefore, based on the data, some specific health components need a special effort, such as oral and general hygiene [72]. In addition, training in talking about such sensitive topics was suggested, such as topics around sexual health [81, 82]. In addition, general training is needed to improve nurses’ understanding of the value of specific health screening items.

All these development ideas are feasible and realistic, but still leave us without a conclusion as to why these good ideas are not realized in daily practice. One reason for this may be nurses’ training needs [83]. For example, in our study, nurses had worked in mental health setting on average for over 20 years and still some health issues, e.g. adverse effects of medication, patients’ difficulties observing physical health concerns and lifestyle typical for patients with SSD, were unclear for nurses [2, 3]. Furthermore, organizational culture can affect nurses’ self-confidence in conducting screenings [84] and our research results revealed that nurses have to prioritize the time used on an appointment between mental health and physical health assessment. Patients with SSD may not have the ability to fill the screening assessment by themself before the appointment [45] and may require the collaboration with a nurse. Moreover, possibilities of using digital technology [85] in physical health screening may be underrated.

### Trustworthiness

We reflected on the trustworthiness of our study in terms of its credibility, dependability, conformability, and transferability [86, 87] as follows. Credibility was confirmed by selecting the context and participants who had different experiences of the topic. By using focus groups and individual interviews in the data gathering, we gained knowledge of various experiences, which increased the possibility of shedding light on the research question from a variety of perspectives [40]. Credibility was further strengthened through presenting the coding process by illustrating how the meaning units from the interviews, extracted codes and categories were produced. The similarities and differences of the research findings are shown with representative quotations from the transcribed text. Dependability was improved through open dialogue among the authors and consistently during the data collection by asking all of the participants similar questions [63]. Conformability was achieved by reporting the research steps carefully. Transferability was increased by presenting a clear and distinct description of the context, recruitment and characteristics of the participants and of the data collection and data analysis.

### Study strengths and limitations

The current study has some limitations that potentially impact the trustworthiness and transferability of the findings. Participants were recruited by a purposive sampling method, which likely caused bias by recruiting those more interested in discussing the topic [88, 89]. Although nurses were trained to understand the meaning of specific inclusion and exclusion criteria for the patients, selection bias may still have occurred in the patient recruitment process and patient data may be biased toward those patients who are more motivated, capable and collaborative to join initiatives. All patient participants were diagnosed with a psychotic disorder (F20–29), but the sample size was relatively small and might limit the transferability of the findings to patients with SSD. Similarly, participants were recruited in one hospital only and due to their narrow ethnic background group, this may also may reduce the transferability of the findings outside Finland.

The qualitative study design itself might have imposed some limitations in several phases during the study. The researcher’s presence during the interviews may have affected the subjects’ responses, even if this is often unavoidable in qualitative research [88, 90]. The researcher conducting the interviews had a deep understanding of the research topic based on her experience in working with persons with SSD. At the same time, having strong pre-assumptions may have caused bias due to a lack of openness to the topic, hence reducing the credibility. Furthermore, it is possible that the short duration of interviews limits the depth of understanding of the topic. Similarly, the small number of nurse participants in some of the focus groups is likely to have limited the potential for productive group discussion. Even though the interviews were conducted individually with patients, it is possible that the patients were hesitant to openly share their views to a person who represents a staff member. Moreover, the transcripts of the digitally recorded interviews were not returned to nurses or patient participants, so member checking of transcripts and categories was not carried out. Formal backtranslation was not conducted for the data, which might also decrease the credibility of the results. Regardless of these limitations, the study has some strengths and consists of rich and informative data regarding the perceptions of nurses and patients.

## Conclusions

Our study results offer a novel diversity of perceptions from nurses and patients toward physical health screening in mental health settings. Patients with schizophrenia spectrum disorders are willing to participate in physical health screening. Although nurses found the HIP-F to be too long, they showed interest in assessing their patients’ physical health and suggested improvements to develop screening to improve its feasibility in clinical practice. Physical health screening should be clear, easy to use and relatively quick. Developing and improving health screening to better suit clinical practice, for example in their length, would further support professionals in conducting and encouraging patients to participate in physical health screening. With this detailed knowledge of perceptions of screening, further research is needed to understand what factors affect the implementation fidelity of physical health screening in clinical mental health practice and to gain an overall understanding on how to improve such implementation.

### Implications

Several studies have emphasized the position of nurses in the assessment of physical health [28, 68, 73]. In order for patients to benefit from the results of physical health assessments in clinical practice, it is crucial that the treatment guidelines are followed, assessment results are available in patient record systems and actions are completed according to health promotion plans. Our findings can be used in supporting professionals to collaborate with patients to participate in physical health screening. Our results are also useful in planning curriculums in nursing education and clinical settings. Finally, our results should encourage nurses to implement regular physical health screenings for patients with SSD followed by appropriate effective health promotion interventions. For effective physical health screening and preventing physical comorbidity and premature deaths, the perceptions explored in our study can be taken into consideration by those who develop screening procedures and health screenings for clinical practice.

## Data Availability

Data generated during and/or analyzed during the study are not publicly available due to ethical restrictions and privacy.

## Abbreviations

BMI: Body mass index
CHIP: Chinese Health Improvement Profile
COREQ: consolidated criteria for reporting qualitative studies
COVID-19: Coronavirus
ECG: electrocardiogram
HIP: Health improvement profile
HIP-F: Finnish health improvement profile
HIV: human immunodeficiency virus
HUH: Helsinki University Hospital
ICD10: 10th revision of the International Classification of Diseases and Related Health Problems
M: Male
MSST: Metabolic Syndrome Screening Tool
Mets: metabolic syndrome
N: nurse
NRC: Nursing Research Center
P: patient
PHC: The physical health check
PhD: Doctorate of Philosophy
QI: quality improvement
RCT: randomized controlled trial
SD: Standard deviation
SSD: schizophrenia spectrum disorder

## References

[1] World Health Organization. Available: https://www.who.int/news-room/fact-sheets/detail/schizophrenia Accessed 12th March 2023.

[2] Filik R, Sipos A, Kehoe P (2006). Cardiovascular and respiratory health of people with schizophrenia. Acta Psychiatrica Scandinavica.

[3] Grover S, Aggarwal M, Dutt A, Chakrabarti S, Avasthi A, Kulhara P, Chauhan N (2012). Prevalence of metabolic syndrome in patients with schizophrenia in India. Psychiatry Res.

[4] Larsen JR, Svensson CK, Vedtofte L, Jakobsen ML, Jespersen HS, Jakobsen MI, Holst JJ (2018). High prevalence of prediabetes and metabolic abnormalities in overweight or obese schizophrenia patients treated with clozapine or olanzapine. CNS Spectr.

[5] Hägg S, Lindblom Y, Mjörndal T, Adolfsson R (2006). High prevalence of the metabolic syndrome among a Swedish cohort of patients with schizophrenia. Int Clin Psychopharmacol.

[6] Dickerson FB, Brown CH, Daumit GL, LiJuan F, Goldberg RW, Wohlheiter K, Dixon LB (2006). Health status of individuals with serious mental illness. Schizophr Bull.

[7] Hughes E, Bassi S, Gilbody S (2016). Prevalence of HIV, hepatitisB, and hepatitis C in people with severe mental illness: asystematic review and meta-analysis. Lancet Psychiatry.

[8] Taquet M, Luciano S, Geddes JR (2020). Bidirectional associa-tions between COVID-19 and psychiatric disorder: retrospec-tive cohort studies of 62354 COVID-19 cases in the USA. Lancet Psychiatry.

[9] Šprah L, Dernovšek MZ, Wahlbeck K, Haaramo P (2017). Psychiatric readmissions and their association with physical comorbidity: a systematic literature review. BMC Psychiatry.

[10] Schizophrenia. Current Care Guidelines. Working group set up by the Finnish Medical Society Duodecim and the Finnish Psychiatric Association. Helsinki: the Finnish Medical Society Duodecim, 2023 (referred February 9, 2023). Available online at: www.kaypahoito.fi Accessed 8th December 2022.

[11] Olfson M, Gerhard T, Huang C (2015). Premature mortalityamong adults with schizophrenia in the United States. JAMAPsychiatry.

[12] Saha S, Chant D, McGrath J (2007). A systematic review of mortalityin schizophrenia: is the differential mortality gap worseningover time?. Arch Gen Psychiatry.

[13] American Psychiatric Association (2004). Practice guideline for the treatment of patients with schizophrenia. Am J Psychiatry.

[14] Department of Health, & Great Britain. Dept. of Health. (2006). Our health, our care, our say: A new direction for community services (Vol. 6737). The Stationery Office.

[15] National Institute for Health and Care Excellence. Psychosis and Schizophrenia in adults: Treatment and Management (NICE Clinical Guideline 178). NICE, 2014. https://www.nice.org.uk/guidance/cg178 Accessed 8th January 2023.

[16] Taylor DM, Barnes TR, Young AH. The Maudsley prescribing guidelines in psychiatry. Wiley; 2021.

[17] Lamontagne-Godwin F, Burgess C, Clement S, Gasston-Hales M, Greene C, Manyande A, Barley E. (2018). Interventions to increase access to or uptake of physical health screening in people with severe mental illness: a realist review. BMJ open, 8(2), e019412. 10.1136/bmjopen-2017-019412 PMC5829934 29440160

[18] Phelan M, Stradins L, Amin D, Isadore R, Hitrov C, Doyle A, Inglis R (2004). The physical health check: a tool for mental health workers. J Mental Health.

[19] Vasudev K, Thakkar PB, Mitcheson N. (2012). Physical health of patients with severe mental illness: an intervention on medium secure forensic unit. Int J Health care Qual Assur. 10.1108/09526861211221527 22755485

[20] Yeomans D, Dale K, Beedle K (2014). Systematic computerized cardiovascular health screening for people with severe mental illness. Psychiatric Bull.

[21] Brunero S, Lamont S (2009). Systematic screening for metabolic syndrome in consumers with severe mental illness. Int J Ment Health Nurs.

[22] Castillo EG, Rosati J, Williams C, Pessin N, Lindy DC (2015). Metabolic syndrome screening and assertive community treatment: a quality improvement study. J Am Psychiatr Nurses Assoc.

[23] Hardy S, White J, Gray R. The Health Improvement Profile: a manual to promote physical wellbeing in people with severe mental illness. M&K Update Ltd; 2015.

[24] McGinty EE, Baller J, Azrin ST (2015). Quality of medical carefor persons with serious mental illness: a comprehensive review. Schizophr Res.

[25] Ilyas A, Chesney E, Patel R (2017). Improving life expectancy in people with serious mental illness: should we place more emphasis on primary prevention?. Br J Psychiatry.

[26] Vancampfort D, Watkins A, Ward PB, Probst M, De Hert M, Van Damme T, Mugisha J (2019). Barriers, attitudes, confidence, and knowledge of nurses regarding metabolic health screening and intervention in people with mental illness: a pilot study from Uganda. Afr Health Sci.

[27] Voort NTVD, Klaessen NC, Poslawsky IE, van Meijel B. (2022). Mental Health nurses’ perceptions of their role in physical screening and lifestyle coaching for patients with a severe Mental illness: a qualitative study. J Am Psychiatr Nurses Assoc, 10783903221085596. 10.1177/10783903221085596 35442098

[28] Happell B, Scott D, Nankivell J, Platania-Phung C (2013). Screening physical health? Yes! But… nurses’ views on physical health screening in mental health care. J Clin Nurs.

[29] Mwebe H (2017). Physical health monitoring in mental health settings: a study exploring mental health nurses’ views of their role. J Clin Nurs.

[30] Butler J, de Cassan S, Turner P, Lennox B, Hayward G, Glogowska M (2020). Attitudes to physical healthcare in severe mental illness; a patient and mental health clinician qualitative interview study. BMC Fam Pract.

[31] Bressington D, Mui J, Wells H, Chien WT, Lam C, White J, Gray R (2016). Refocusing on physical health: Community psychiatric nurses’ perceptions of using enhanced health checks for people with severe mental illness. Int J Ment Health Nurs.

[32] White J, Gray R, Jones M (2009). The development of the serious mental illness physical Health Improvement Profile. J Psychiatr Ment Health Nurs.

[33] Hardy S, Deane K, Gray R (2012). The Northampton Physical Health and Wellbeing Project: the views of patients with severe mental illness about their physical health check. Mental Health Family Med.

[34] Bressington D, Chien WT, Mui J, Lam KKC, Mahfoud Z, White J, Gray R (2018). The Chinese health improvement profile (CHIP) for people with severe mental illness: a cluster randomized controlled trial. Int J Ment Health Nurs.

[35] Thongsai S, Gray R, Bressington D (2016). The physical health of people with schizophrenia in Asia: baseline findings from a physical health check programme. J Psychiatr Ment Health Nurs.

[36] White J, Lucas J, Swift L, Barton GR, Johnson H, Irvine L, Gray RJ (2018). Nurse-facilitated health checks for persons with severe mental illness: a cluster-randomized controlled trial. Psychiatric Serv.

[37] Werkkala C, Välimäki M, Anttila M, Pekurinen V, Bressington D (2020). Validation of the Finnish Health Improvement Profile (HIP) with patients with severe mental illness. BMC Psychiatry.

[38] Fleuren MAH, Wiefferink CH, Paulussen TGWM (2004). Determinants of innovation within health care organizations: literature review and Delphi-study. Int J Qual Health Care.

[39] Dickens GL, Ion R, Waters C, Atlantis E, Everett B (2019). Mental health nurses’ attitudes, experience, and knowledge regarding routine physical healthcare: systematic, integrative review of studies involving 7,549 nurses working in mental health settings. BMC Nurs.

[40] Renwick L, Drennan J, Sheridan A, Lyne J, Kinsella A, O’Callaghan E, Clarke M (2019). Disagreement between service-users and clinicians assessment of physical health during early psychosis. Early Interv Psychiat.

[41] Patton MQ. Qualitative Research & Evaluation Methods. 3rd edition. London: SAGE Publications; 2002.

[42] Sandelowski M (2000). Focus on research methods: whatever happened to qualitative description. Res Nurs Health.

[43] Hunter D, McCallum J, Howes D. (2019). Defining exploratory-descriptive qualitative (EDQ) research and considering its application to healthcare. J Nurs Health Care, 4(1).

[44] Morgan DL. Focus groups as qualitative research. Volume 16. Sage; 1996.

[45] Gold JM, Harvey PD (1993). Cognitive deficits in schizophrenia. Psychiatr Clin North Am.

[46] Telford R, Faulkner A. (2004). Learning about service user involvement in mental health research. J Mental Health, 13(6).

[47] Kleiman S. (2004). Phenomenology: to wonder and search for meanings. Nurse Res, 11(4). 10.7748/nr2004.07.11.4.7.c6211 15227895

[48] Tong A, Sainsbury P, Craig J (2007). Consolidated criteria for reporting qualitative research (COREQ): a 32-item checklist for interviews and focus groups. Int J Qual Health Care.

[49] HUS Psychiatry. 2023. https://www.hus.fi/en/search?q=psychiatry&size=n_20_ Accessed 14th November 2022.

[50] World Health Organization. ICD-10: international statistical classification of diseases and related health problems: tenth revision. 2004. https://apps.who.int/iris/bitstream/handle/10665/42980/9241546530_eng.pdf?sequence=1&isAllowed=y Accessed 16th November 2022.

[51] HUS Psychiatry. 2023. https://www.hus.fi/en/patient/treatments-and-examinations/psychoses Accessed 14th November 2022.

[52] Hennink MM, Kaiser BN, Weber MB (2019). What influences saturation? Estimating sample sizes in focus group research. Qual Health Res.

[53] Giacomini MK, Cook DJ, Evidence-Based Medicine Working Group. Evidence-Based Medicine Working Group, &. (2000). Users’ guides to the medical literature: XXIII. Qualitative research in health care A. Are the results of the study valid? *Jama*, *284*(3), 357–362. 10.1001/jama.284.3.357 10891968

[54] Côté L, Turgeon J (2005). Appraising qualitative research articles in medicine and medical education. Med Teach.

[55] Grove. SK, Burns N, Gray. J. 2013. The Practice of Nursing Research; Appraisal, Synthesis, and Generation of Evidence. 7th edition. W.B. Saunders Comp., Philadelphia.

[56] Guest G, Bunce A, Johnson L (2006). How many interviews are enough? An experiment with data saturation and variability. Field Methods.

[57] Gill P, Stewart K, Treasure E, Chadwick B (2008). Methods of data collection in qualitative research: interviews and focus groups. Br Dent J.

[58] Elo S, Kyngäs H (2008). The qualitative content analysis process. J Adv Nurs.

[59] Krueger RA. Focus groups: a practical guide for applied research. Sage; 2014.

[60] Maharaj N (2016). Using field notes to facilitate critical reflection. Reflective Pract.

[61] Malterud K, Siersma VD, Guassora AD (2016). Sample size in qualitative interview studies: guided by information power. Qual Health Res.

[62] Green J, Thorogood N. (2004). Qualitative Research Methods.

[63] Vaismoradi M, Turunen H, Bondas T (2013). Content analysis and thematic analysis: implications for conducting a qualitative descriptive study. Nurs Health Sci.

[64] Lambert SD, Loiselle CG (2008). Combining individual interviews and focus groups to enhance data richness. J Adv Nurs.

[65] Graneheim UH, Lundman B (2004). Qualitative content analysis in nursing research: concepts, procedures and measures to achieve trustworthiness. Nurse Educ Today.

[66] Polit DF, Beck CT. Nursing research: principles and methods. Lippincott Williams & Wilkins; 2004.

[67] Lincoln Y. S., Guba E. G. However, is it rigorous? Trustworthiness and authenticity in naturalistic evaluation. New Dir Program Evaluation. 1986;1986(30):73–84.

[68] Happell B, PLATANIA-PHUNG C, Gray R, Hardy S, Lambert T, McAllister M, Davies C (2011). A role for mental health nursing in the physical health care of consumers with severe mental illness. J Psychiatr Ment Health Nurs.

[69] Shuel F, White J, Jones M, Gray R (2010). Using the serious mental illness health improvement profile [HIP] to identify physical problems in a cohort of community patients: a pragmatic case series evaluation. Int J Nurs Stud.

[70] Brunero S, Lamont S (2010). Health behavior beliefs and physical health risk factors for cardiovascular disease in an outpatient sample of consumers with a severe mental illness: a cross-sectional survey. Int J Nurs Stud.

[71] Campion G, Francis V, Preston A, Wallis A, Lincoln N (2005). Health behavior and motivation to change. Mental Health Nurs.

[72] Çelik Ince S, Partlak Günüşen N, Serçe Ö (2018). The opinions of Turkish mental health nurses on physical health care for individuals with mental illness: a qualitative study. J Psychiatr Ment Health Nurs.

[73] Blanner Kristiansen C, Juel A, Vinther Hansen M, Hansen AM, Kilian R, Hjorth P (2015). Promoting physical health in severe mental illness: patient and staff perspective. Acta Psychiatrica Scandinavica.

[74] Kaltiala-Heino R, Välimäki M, Korkeila J, Tuohimäki C, Lehtinen V (2003). Involuntary medication in psychiatric inpatient treatment. Eur Psychiatry.

[75] de Leon J, Verghese C, Tracy JI, Josiassen RC, Simpson GM (1994). Polydipsia and water intoxication in psychiatric patients: a review of the epidemiological literature. Biol Psychiatry.

[76] Graham KL, Carson CM, Ezeoke A, Buckley PF, Miller BJ (2014). Urinary tract infections in acute psychosis. J Clin Psychiatry.

[77] Miller BJ, Graham KL, Bodenheimer CM, Culpepper NH, Waller JL, Buckley PF (2013). A prevalence study of urinary tract infections in acute relapse of schizophrenia. J Clin Psychiatry.

[78] Warnier RMJ, van Rossum E, Du Moulin MFMT, van Lottum M, Schols JMGA, Kempen GIJM (2021). The opinions and experiences of nurses on frailty screening among older hospitalized patients. An exploratory study. BMC Geriatr.

[79] Happell B, Platania-Phung C (2015). Cardiovascular health promotion and consumers with mental illness in Australia. Issues Ment Health Nurs.

[80] Robson D, Haddad M, Gray R, Gournay K (2013). Mental health nursing and physical health care: a cross-sectional study of nurses’ attitudes, practice, and perceived training needs for the physical health care of people with severe mental illness. Int J Ment Health Nurs.

[81] Quinn C, Platania-Phung C, Bale C, Happell B, Hughes E (2018). Understanding the current sexual health service provision for mental health consumers by nurses in mental health settings: findings from a survey in Australia and England. Int J Ment Health Nurs.

[82] Quinn C, Happell B, Browne G (2012). Opportunity lost? Psychiatric medications and problems with sexual function: a role for nurses in mental health. J Clin Nurs.

[83] Blythe J, White J (2012). Role of the mental health nurse toward physical health care in serious mental illness: an integrative review of 10 years of UK literature. Int J Ment Health Nurs.

[84] Tzeng WC, Su PY, Yeh SH, Chang TW, Lin CH, Feng HP (2023). Nurses’ views on the provision of physical healthcare for individuals with comorbid mental illness and chronic disease. Int J Ment Health Nurs.

[85] Chivilgina O, Elger BS, Jotterand F (2021). Digital Technologies for Schizophrenia Management: a descriptive review. Sci Eng Ethics.

[86] Polit DF, Beck CT, Polit F. Cheryl Tatano Beck-Details-Trove; Wolters Kluwer Health/Lippincott Williams & Wilkins: Philadelphia, PA, USA, 2010; 301–7.

[87] Stalmeijer RE, McNaughton N, Van Mook WNKA (2014). Using focus groups in medical education research: AMEE Guide 91. Med Teach.

[88] Firth J, Rosenbaum S, Stubbs B, Gorczynski P, Yung AR, Vancampfort D. Motivating factors and barriers toward exercise in severe mental illness: a systematic review and meta-analysis. Psychol Med. 2016;46(14):2869–81. 10.1017/S0033291716001732 PMC5080671 27502153

[89] Mulder CL, Jochems E, Kortrijk HE. The motivation paradox: higher psychosocial problem levels in severely mentally ill patients are associated with less motivation for treatment. Soc Psychiatry Psychiatr Epidemiol. 2014;49(4):541–8. 10.1007/s00127-013-0779-7 24136001

[90] World Medical Association (2001). World Medical Association Declaration of Helsinki. Ethical principles for medical research involving human subjects. Bull World Health Organ.

[91] FINLEX 523/1999. Finnish Personal Data Act. Ministry of Social Affairs and Health, Finland. 2001. https://www.finlex.fi/fi/laki/kaannokset/1999/en19990523.pdf. Accessed 28 Jan 2023.

[92] World Medical Associationon. World medical association declaration of Helsinki. Ethical principles for medical research involving human subjects. Bull World Health Organ. 2001;9(4):373. PMC2566407 11357217

