# Correction: Nurses’ and patients’ perceptions of physical health screening for patients with schizophrenia spectrum disorders: a qualitative study

**DOI:** 10.1186/s12912-024-02037-1

**Published:** 2024-06-03

**Authors:** Camilla Långstedt, Daniel Bressington, Maritta Välimäki

**Affiliations:** 1 Faculty of Medicine, Department of Nursing, University of Turku, Kiinamyllynkatu 10, Medisiina B, Turku, 20520 Finland; 2 Professor in Mental Health, Faculty of Health, Charles Darwin University, Casuarina, Australia; 3 Faculty of Nursing, Chiang Mai University, 110/406 Inthawaroros Road, Sri Phum District, Chiang Mai, Thailand; 4 School of Public Health, University of Helsinki, Helsinki, Finland

**Correction to: BMC Nursing (2024) 23:321**

10.1186/s12912-024-01980-3

Following publication of the original article [1], the authors reported an error in the order of author names, the given names and the family names have been mistakenly swapped.

The correct author names have been provided in this Correction and the original article [1] has been corrected.
